# Changes in chlamydia prevalence and duration of infection estimated from testing and diagnosis rates in England: a model-based analysis using surveillance data, 2000–15

**DOI:** 10.1016/S2468-2667(18)30071-9

**Published:** 2018-06-05

**Authors:** Joanna Lewis, Peter J White

**Affiliations:** a National Institute for Health Research Health Protection Research Unit in Modelling Methodology and Medical Research Council Centre for Outbreak Analysis and Modelling, Department of Infectious Disease Epidemiology, Imperial College London, UK; b Modelling and Economics Unit, National Infection Service, Public Health England, London, UK

## Abstract

**Background:**

Chlamydia screening programmes have been implemented in several countries, but the effects of screening on incidence, prevalence, and reproductive sequelae remain unclear. In England, despite increases in testing with the rollout of the National Chlamydia Screening Programme (NCSP; 2003–08), prevalence estimated in 10-yearly population-based surveys was similar before (1999–2001) and after (2010–12) the programme. However, the precision of these previous estimates was limited by the low numbers of infections. We aimed to establish annual, rather than 10-yearly, estimates of chlamydia prevalence and infection duration.

**Methods:**

In this model-based analysis, we used previously published minimum and maximum estimates and Public Health England data for chlamydia test coverage and diagnoses in men and women aged 15–24 years in England, before, during, and after the scale-up of national chlamydia screening. We used a mechanistic model, which accounted for symptomatic chlamydia testing and asymptomatic screening, to estimate changes in prevalence and average duration of infections for each year. We describe estimates derived from the maximum and minimum numbers of tests and diagnoses as maximum and minimum estimates, regardless of their relative magnitude.

**Findings:**

The data included numbers of tests and diagnoses in men and women aged 15–19 years and 20–24 years in England each year from 2000 to 2015. We estimated reductions in prevalence and average infection duration in both sexes once screening was fully implemented. From 2008 to 2010, estimated posterior median prevalence reductions in people aged 15–24 years were 0·68 percentage points (95% credible interval 0·26–1·40; minimum) and 0·66 percentage points (0·25–1·37; maximum) for men and 0·77 percentage points (0·45–1·27) for women (minimum and maximum estimates were the same for women). Over the same time period, mean duration of infection reduced by 75 days (95% credible interval 17–255; minimum) and 74 days (95% credible interval 17–247; maximum) in men and 30 days (22–40) in women. Since 2010, some of the progress made by the NCSP has been reversed, alongside a reduction in testing.

**Interpretation:**

Our analysis provides the first evidence for a reduction in chlamydia prevalence in England concurrent with large-scale population testing. It also shows a consistent decline in the average duration of infections, which is a measure of screening effectiveness that is unaffected by behavioural changes.

**Funding:**

National Institute for Health Research, Medical Research Council.

## Introduction

Sexually transmitted infections such as chlamydia are thought to be an important cause of infertility worldwide, and WHO estimates that there are 131 million new chlamydia infections each year in total (including undetected cases).^1^ Their Global Health Sector Strategy on Sexually Transmitted Infections recommends chlamydia prevalence monitoring in high-risk groups, including adolescents.^1^ Other studies have recommended definition of acceptable local targets for chlamydia prevalence; reduction of chlamydia infections to reduce the incidence of pelvic inflammatory disease (PID);^2^ and establishment of surveillance systems to investigate the effects of control policies on PID and its complications.^3^ However, prevalence monitoring is challenging because most chlamydia infections are asymptomatic^4^ and therefore not detected by syndromic case reporting systems. WHO and the European Centre for Disease Prevention and Control (ECDC) both note that the best strategies to control and monitor chlamydia infections have yet to be established, and they encourage further research into these areas.^1^, ^5^

Programmes of screening or widespread testing for chlamydia form part of national sexual health provision in several countries around the world.^5^, ^6^ Early modelling studies predicted that screening would be highly effective, but more recent studies have been more conservative and have increasingly considered the role of partner notification as well as widespread testing.^7^ A 2016 Cochrane review^8^ highlighted a controlled trial in the Netherlands that found low screening uptake and no change in the proportion of tested individuals who were positive for chlamydia (ie, positivity) after three annual screening invitations; whereas by contrast, a trial in female sex workers in Peru found a reduction in prevalence after 4 years. In the USA, the 2-yearly cycles of surveys for the NHANES studies indicated a decrease in chlamydia prevalence from 1999 to 2008 in people aged 14–39 years overall, but no change in women aged 15–25 years, which is the population targeted for routine screening.^9^ Overall prevalence remained similar over the three cycles from 2007 to 2012.^10^

**Research in context**

**Evidence before this study**

Widespread chlamydia screening has been implemented in several countries, but the extent of its effects is unclear. A 2016 Cochrane review investigating the effects of chlamydia screening searched the Cochrane Sexually Transmitted Infections Group Specialised Register and other registries with the terms (variations on and synonyms of) “genital chlamydia infection” and “screening” to Feb 14, 2016. The authors searched for randomised controlled trials (RCTs) done in adults (people older than 13 years), which compared a chlamydia screening intervention with usual care and reported one of the following as a primary outcome: chlamydia prevalence; pelvic inflammatory disease in women; epididymitis in men; or incidence of preterm delivery. The review found two trials investigating the effect of chlamydia screening on population chlamydia prevalence. A controlled trial in the Netherlands found low screening uptake and no change in chlamydia positivity after three annual screening invitations, whereas a trial in female sex workers in Peru found a reduction in prevalence after 4 years. Both trials were assessed as providing low-quality evidence. England's extensive National Chlamydia Screening Programme (NCSP) was rolled out between 2003 and 2008, but population-based surveys in 1999–2001 and 2010–12 found little change in chlamydia prevalence in young people, although confidence intervals were wide. Comprehensive estimates of annual numbers of chlamydia tests and diagnoses in young people in England from 2000 to 2015, by sex and age group, have recently become available. However, no study has shown how these data correspond to changes in chlamydia prevalence.

**Added value of this study**

In our study, we provide a more-detailed picture of the year-to-year changes in chlamydia prevalence before, during, and after the period over which the NCSP was rolled out and test coverage increased (2003–08). We estimated prevalence by age group and sex each year from 2000 to 2015 using a recently developed evidence synthesis method and newly published data for test coverage and numbers of diagnoses, combined with information on natural history and care-seeking behaviour. Additionally, we estimated the average duration of infection, which clearly declined year-on-year in both sexes as screening activity increased, and particularly after full-scale implementation of NCSP. Since 2010, rates of testing and diagnosis have reduced, and modest increases in inferred prevalence and the average duration of infection have occurred in all sex and age groups assessed.

**Implications of all the available evidence**

Our analysis provides evidence for a reduction in chlamydia prevalence and average duration of infection in England associated with large-scale population screening, which occurred in both sexes. Reduction in average duration of infection is the better indicator of screening programme success because prevalence is also affected by changes in population sexual risk behaviour. Our evidence synthesis using data from a national testing programme complements existing data from clinical trials to improve understanding of the effects of chlamydia screening. Declines in testing in England since 2010, alongside partial reversals of health gains, are concerning.

In England, the National Chlamydia Screening Programme (NCSP) has offered chlamydia testing to men and women aged 15–24 years since 2003, with full nationwide implementation completed in 2008. Screening is commissioned locally, and offered in settings including by general practitioners, sexual health services, pharmacies, and online. In 2016, 20·7% of the eligible population was tested and 9·1% of tests were positive, corresponding to a national detection rate of 1882 per 100 000 people aged 15–24 years. However, chlamydia prevalence in young people was similar between population-based prevalence surveys done in 1999–2001 (National Surveys of Sexual Attitudes and Lifestyles [Natsal]-2) and in 2010–12 (Natsal-3).^11^ Prevalence in women aged 18–24 years was 3·1% (95% CI 1·8–5·2) in 1999–2001 and 3·2% (2·2–4·6) in 2010–12. The corresponding estimates for men were 2·9% (1·3–6·3) in 1999–2001 and 2·6% (1·7–4·0) in 2010–12.^11^ Surveys done once per decade provide little information on prevalence trends, and over time, marked changes have occurred in uptake of chlamydia testing in England.

Surveillance data provide more detailed information on trends, but no system in England had recorded complete annual numbers of chlamydia tests and diagnoses by sex and age until the Chlamydia Testing Activity Dataset became available in 2012. However, a study published in 2017, which combined data from several surveillance systems, has provided estimates for these quantities before 2012.^12^ In this analysis we use a model-based framework to estimate changes in chlamydia prevalence in England each year from 2000 to 2015 as diagnostic test coverage changed before, during, and after the rollout of the NCSP.

## Methods

### Study design and data sources

This study was a modelling analysis that used previously published estimates and Public Health England data. Numbers of chlamydia tests and diagnoses in England for the period 2000–12 were estimated by Chandra and colleagues,^12^ who reported minimum and maximum estimates. For 2013 onwards, comprehensive data have been published by Public Health England.^13^ Our definition of testing coverage is that used by the NCSP: the annual number of chlamydia tests divided by the number of sexually active individuals in the relevant age and sex group. Mid-year population estimates came from the Office for National Statistics.^14^

We estimated chlamydia prevalence and incidence in England in men and women aged 15–24 years from 2000 to 2015 using a method that synthesises data on chlamydia testing and diagnosis with information on care-seeking behaviour and infection natural history. Details of the method are published elsewhere.^15^ We did analyses separately by sex and age group (men and women, ages 15–19 years and 20–24 years). For 2000–12, we did separate calculations using the minimum and maximum testing and diagnosis estimates.^12^

### Statistical analysis

The method is based on a model of a closed population at steady state, in which uninfected individuals who become infected move into either a symptomatic-infected or an asymptomatic-infected state. Symptomatic individuals seek treatment, whereas asymptomatic infections are detected through asymptomatic testing programmes. By allowing for different rates of testing in individuals who are symptomatic and non-symptomatic (either uninfected or with an asymptomatic infection), the model accounts for the changing proportion of tests done in each of these groups. We used data from previous studies to parameterise natural history and care-seeking behaviour (appendix), which remain the same from year to year. The observed test coverage and annual diagnoses per capita, which typically change from year to year, can then be used to estimate the proportion of the population in each state, and hence the chlamydia prevalence. The strength of this method is that it provides an estimate of prevalence, rather than relying on proxies such as diagnoses (which are affected by the amount of testing) or positivity (which is affected by the risk profile of those tested).

Prevalence estimates were then used to calculate estimated year-on-year prevalence changes in each sex. For each sampled set of natural history parameters, for each year, the prevalence sample for the previous year was subtracted from the sample for the current year to give a sample for the change in prevalence. Together, the samples provide a posterior distribution for the year-to-year change.

Because our method is based on a mechanistic model, it can also be used to establish other quantities, including the average duration of infections. The average duration of infections was calculated by dividing prevalence by incidence.

All computation and visualisation of results was done in the Python language (version 2.7.10), and results and figures can be reproduced using the code available online.

### Role of the funding source

The funders of this study had no role in study design, data collection, analysis, or interpretation, or writing of the report. The corresponding author had full access to all the data in the study and the final responsibility to submit for publication.

## Results

**Figure 1:**
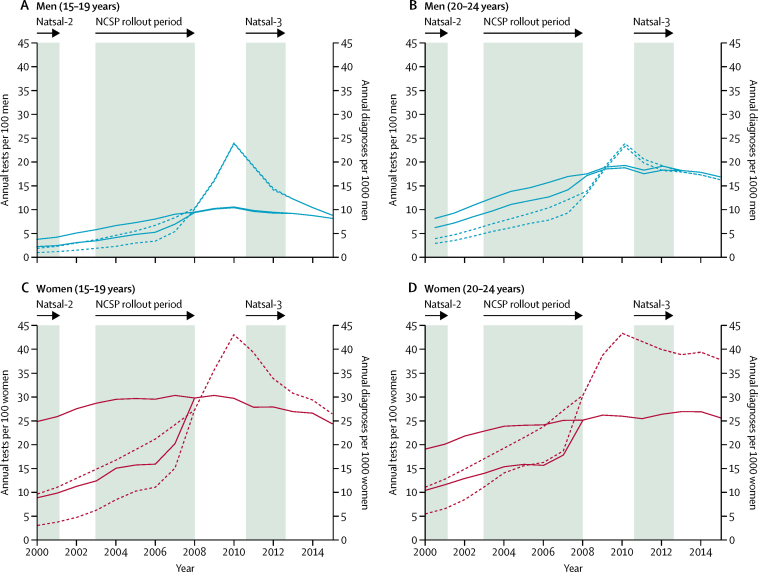
Chlamydia tests and diagnoses in young people in England, 2000–15 (A) Men aged 15–19 years; (B) men aged 20–24 years; (C) women aged 15–19 years; and (D) women aged 20–24 years. Dotted lines show tests; solid lines show diagnoses. Pairs of lines show minimum and maximum numbers of tests and diagnoses. Data up to 2012 are from Chandra and colleagues;^12^ data from 2013 are from Public Health England.^13^

Chlamydia test coverage increased in both sexes and age groups every year from 2000 to 2010, with both the maximum and the minimum test numbers (figure 1). From 2010 onwards, coverage decreased in all age and sex groups, but the decrease was greater in people aged 15–19 years than those aged 20–24 years. In men, diagnoses per capita increased until 2010, after which point they stayed at a similar level or began to decrease slightly (figure 1A, B). The trend of diagnoses in women was less certain than in men, but increased until around 2008 (figure 1C, D). In women aged 15–19 years, the diagnoses per capita began to decrease again after 2010; whereas in those aged 20–24 years, diagnoses remained fairly constant from 2009 onwards.

**Figure 2:**
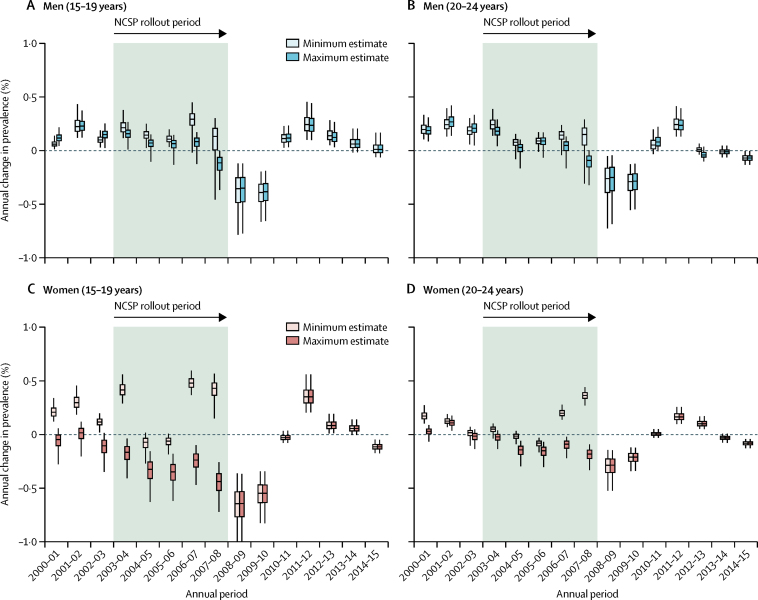
Inferred annual changes in chlamydia prevalence in young people in England, 2000–15 (A) Men aged 15–19 years; (B) men aged 20–24 years; (C) women aged 15–19 years; and (D) women aged 20–24 years. Each datapoint shows the change between estimates for one year and those for the subsequent year. For the later years the minimum and maximum estimates were identical so boxes show the same results. Coloured boxes show the 50% credible interval, the small horizontal lines show the median estimate, and whiskers show the 95% credible interval.

In men, the model estimated that chlamydia prevalence was increasing in the years immediately preceding the introduction of the NCSP (figure 2A, B). The rollout of the NCSP coincided with a reversal of this trend and by 2009, when the programme was fully implemented nationally, the model estimated that prevalence was decreasing. In men aged 15–24 years, the prevalence decreases over the 2 years after full completion of NCSP (2008–10; appendix), estimated using minimum and maximum testing and diagnosis estimates, were 0·68 percentage points (95% credible interval 0·26–1·40; minimum) and 0·66 percentage points (0·25–1·37; maximum). However, prevalence increased in 2010–12, followed by only minor estimated changes in the years after (figure 2A, B).

The pattern of annual estimated prevalence changes in women was less clear than in men (figure 2C, D). Incomplete data collection before 2008^12^ led to major uncertainty in the numbers of tests and diagnoses, making it difficult to say whether prevalence was increasing or decreasing year-on-year before and during the NCSP rollout. Following full implementation in 2008, however, the pattern was almost identical to that in men: an initial estimated reduction in prevalence, which was reversed in 2011–12, then followed by only minor estimated change. In women aged 15–24 years, the estimated decrease in prevalence over the period 2008–10 was 0·77 percentage points (0·45–1·27)—maximum and minimum test and diagnosis numbers were the same for women aged 15–24 years after 2008.

We compared prevalence estimated from surveillance data in 2000 and 2011 to the survey-based estimates from Natsal, and the estimates were in agreement (appendix).

**Figure 3:**
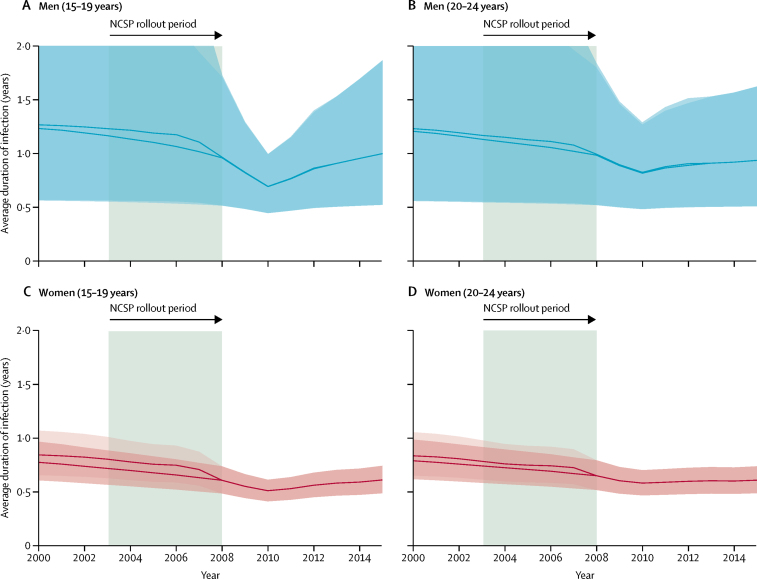
Median absolute estimated duration of chlamydia infection in young people in England, 2000–15 (A) Men aged 15–19 years; (B) men aged 20–24 years; (C) women aged 15–19 years; and (D) women aged 20–24 years. Shading shows 95% credible interval for estimated median duration of infection. The two lines and shading show estimates from the minimum and maximum numbers of tests and diagnoses; dark shading shows overlap of minimum and maximum.^12^

Our results showed a decrease in the estimated median durations of infections in both sexes, with reductions every year, 2000–10 (figure 3). These estimated reductions began before the introduction of the NCSP and continued throughout and after its rollout until 2010, as rates of testing increased. In men aged 15–24 years, the posterior median decreases in the duration of infection from 2008 to 2010, estimated using the minimum or maximum testing and diagnosis figures, were 75 days (95% credible interval 17–255; minimum) and 74 days (17–247; maximum). In women aged 15–24 years, the decrease was 30 days (22–40). However, testing declined after 2010 and the estimated average duration of infections increased in turn. The estimated average duration of infections was in general longer in men than in women, and had greater uncertainty, because of a longer and less-certain duration of untreated infections in men.^16^ However, changes in average duration (data not shown) had less uncertainty than the absolute duration estimates (figure 3), and clear evidence supported the direction of change, showing annual reductions in 2000–10, followed by increases in 2010–15.

## Discussion

Our analysis suggests that in both men and women, chlamydia prevalence and the average duration of infections fell during the period immediately following the full-scale implementation of the NCSP in 2008. However, after 2010 our analysis estimated that prevalence increased and then stabilised in both sexes, whereas duration of infection increased—slightly in women, and substantially in men.

The best population-based estimates for chlamydia prevalence in England come from the Natsal-2 survey of 1999–2001 and the Natsal-3 survey of 2010–12.^11^, ^17^ Comparison of these surveys found little evidence of any prevalence change, although small numbers of infections meant the CIs were wide. Our results in people aged 15–19 years and 20–24 years are consistent with these estimates but add to the picture by using annual figures from surveillance data to examine estimated changes in prevalence in the years between the surveys, and since Natsal-3.

Testing and diagnoses increased in men in the years between the two Natsal surveys, and we estimated annual increases in prevalence followed by declines. Increases before 2004 were driven in our analysis by increasing incidence, probably due to behavioural changes. Rising prevalence could have contributed to the increased testing, with more infected men leading to more symptomatic men seeking tests. The later declines in prevalence coincided with the full-scale implementation of the NCSP. In women before 2008, the large differences between minimum and maximum estimates of numbers of tests and diagnoses make the trend in estimated changes in prevalence unclear, but clear estimated reductions in prevalence followed full-scale implementation of the NCSP in 2008. In women, the prevalence changes estimated using the maximum estimates of tests and diagnoses have a more plausible trajectory than those from the minimum estimates, showing a less abrupt change in testing and diagnoses from 2007 to 2008 and more consistent changes in prevalence. These estimates suggest that prevalence fell as testing increased. Absolute changes were small (<1%), but nonetheless important as a proportion of prevalence, which was also low (around 2–3% in men and women aged 18–24 years in both Natsal-2^17^ and Natsal-3^11^).

In both men and women, the greatest estimated reductions in prevalence were between 2008 and 2010. This period corresponded to the greatest annual increases in testing. Using the maximum estimates for chlamydia testing and diagnosis rates, the most marked estimated increases in prevalence occurred in 2011–12. This corresponded to a decrease in testing in all four age and sex groups.

Attempting to assess the performance of a chlamydia screening programme through changes in prevalence is difficult because of confounding effects of changes in sexual behaviour. Increases in risky behaviour would attenuate reductions in prevalence, while reductions in risky behaviour would exaggerate the prevalence reductions caused by screening. Regardless of changes in sexual behaviour, a successful screening programme would reduce the average duration of infections—thereby reducing prevalence, incidence of onward transmission, and incidence of sequelae to lower levels than would have occurred without screening. We estimated a continuous decline in the average duration of infection in both sexes from 2001 to 2010 because of increased asymptomatic testing, despite estimated increases and then decreases in prevalence during that time. Based on estimated average duration of infection, the NCSP appears to have been successful, although we note some reversal of progress in the years since 2010, coinciding with declining rates of testing. The decline in infection duration before the NCSP is probably due to increasing awareness of chlamydia by clinicians leading to the increase in testing and diagnosis ahead of the official implementation of NCSP in 2003–08, with national screening having been formally considered from 1998 and pilot studies done since 1999.^18^

We estimated a longer duration of infection in men than women, partly due to slower natural clearance of untreated, asymptomatic infections in men than women,^16^ and partly due to less frequent asymptomatic screening in men than women. These two factors outweigh the larger proportion of incident infections in men than in women that are symptomatic and prompt testing.

Additional work on the natural history and pathophysiology of chlamydia infection would complement improved surveillance to provide a better understanding of the short-term and long-term effects of chlamydia screening on population sexual and reproductive health.^2^, ^3^, ^15^, ^16^, ^19^ Good evidence supports the idea that chlamydia infection and pelvic inflammatory disease increase a woman's risk of future reproductive morbidity.^2^ Reproductive complications might take many years to occur, depending on when a woman becomes, or attempts to become, pregnant. As time goes on, however, data on reproductive morbidity could complement analysis of testing and diagnosis data. Some concerns exist that chlamydia screening and treatment might lead to more repeat infections, potentially increasing risk of reproductive morbidity compared with a single, untreated infection.^3^ However, whether the higher risk associated with repeat infections^2^ is due to increased exposure time or an increased per-episode risk is unknown. No data on repeat infections is available to investigate this hypothesis further. Chlamydia might induce some degree of immunity to future infections,^3^ but this would not affect any of our estimates because our methods estimate the average force of infection across all uninfected people that reproduces the recorded annual numbers of tests and diagnoses.

The precision of our estimates of infection duration is limited by uncertainty in chlamydia's natural history,^15^ particularly the clearance rate of untreated infections in men,^16^ and the proportion of infections that become symptomatic in both sexes. However, these uncertainties have less of an effect on estimated changes in prevalence and duration of infection than on absolute estimates. Similarly, posterior distributions for both prevalence and the average duration of infection are insensitive to changes in symptomatic treatment seeking (appendix).

Uncertainty also exists in the numbers of chlamydia tests and diagnoses before 2012, particularly in women.^12^ The true values for tests and diagnoses in women are probably closer to the maximum estimates, since the minimum estimates have an abrupt transition in 2007–08, and the maximum estimates provide a smoother pattern of prevalence changes in the years 2000–10, which has fewer turning points and is closer to the pattern in men. Correlations have been reported between infection risk factors and testing behaviour,^20^ which are not formally accounted for in our estimation approach because they are not recorded in the surveillance data, but sensitivity analysis has indicated that these have a very small effect on prevalence estimates.^15^

With constrained public health budgets worldwide, more-robust assessments of chlamydia screening programme performance are needed urgently. Population-based surveys such as Natsal^11^, ^17^ are valuable but can only be done infrequently, which limits the information they can provide to assess the performance of chlamydia screening programmes. The partial reversal of screening benefits in England since 2010, associated with a reduction in testing activity, points towards a need to maintain screening to maintain control of infection. A combination of effective prevention (eg, condom use promotion), case-finding, and treatment, including both screening and partner notification and management, are needed for effective chlamydia control. With improved surveillance data, the efficiency of screening might be increased by better targeting of those at greatest risk of infection, to reduce the volume of testing required to achieve a similar diagnosis rate and to address health inequalities. Ultimately, planning a broad and effective strategy for chlamydia control requires health economic analysis, considering the cost of the different options as well as their effectiveness. Before the introduction of NCSP, detailed mathematical and economic modelling was done;^21^ now, with more than a decade of data since the implementation of NCSP, we recommend revisiting these models to plan the most cost-effective chlamydia control strategies, incorporating prevention, screening, and partner notification.

We advocate monitoring of test coverage and diagnoses, which can be used to estimate chlamydia prevalence, incidence, duration of infection and changes in these quantities with the method we have used in this study. The method^15^ can readily incorporate improved surveillance data (eg, recording whether the infection was symptomatic, the duration of any symptoms, why the patient was tested, and information on risk behaviour) to increase the precision of estimates and compare performance in different social groups to assess inequalities. Additionally, we endorse the recommendation for the monitoring of chlamydia-related complications, including PID.^3^ Other causes of PID, such as gonorrhoea infection, could also be included in the analysis subject to the required data being collected.

In summary, this study provides evidence that increased chlamydia testing in England has reduced the prevalence of chlamydia infection and the average duration of infections in both men and women, but that these benefits have partly reversed since 2010. This evidence supports chlamydia screening as a control strategy, in conjunction with other measures, including effective partner notification.

For more **information on the Chlamydia Testing Activity Dataset** see https://www.gov.uk/guidance/chlamydia-testingactivity-dataset-ctad

For the **code to reproduce the results and figures from this study** see https://github.com/joanna-lewis/ct_trends
