# Supplementary material for: Changes in chlamydia prevalence and duration of infection estimated from testing and diagnosis rates in England: a model-based analysis using surveillance data, 2000–15
Source: Lancet Public Health. 2018 Jun 5;3(6):e271–8. doi: 10.1016/S2468-2667(18)30071-9 (PMC5990491; doi:10.1016/S2468-2667(18)30071-9)
Supplement: Supplementary appendix [file mmc1.pdf]

THE LANCET
Public Health

Supplementary appendix

This appendix formed part of the original submission and has been peer reviewed. We post it as supplied by the authors.

Supplement to: Lewis J, White PJ. Changes in chlamydia prevalence and duration of infection estimated from testing and diagnosis rates in England: a model-based analysis using surveillance data, 2000–15. *Lancet Public Health* 2018; published online May 15. http://dx.doi.org/10.1016/S2468-2667(18)30071-9.

Changes in chlamydia prevalence and duration of infection inferred from testing and diagnosis rates in England: an evidence synthesis using surveillance data, 2000-2015: Appendix

Joanna Lewis and Peter J. White

Parameter Description	Population		α	β	Mean (95% range: (2.5^{th}, 97.5^{th}) centiles)	Reference
Test performance: sensitivity; probability of a positive result, given infected	men		33	1	0.971 (0.894, 0.999)	[1]
	women		130	13	0.909 (0.857, 0.950)	[2]
Test performance: (1-specificity); probability of a positive result, given uninfected	men		3	951	0.00314 (0.00065, 0.00756)	[1]
	women		5	2324	0.00215 (0.00070, 0.00439)	[2]
Rate of spontaneous recovery ($year^{-1}$)	men		Distribution sampled using Markov Chain Monte Carlo methods.		0.470 (0.059, 1.274)	[3-11]
	women				0.728 (0.591, 0.874)	[3,6-9,12-16]
Rate of treatment seeking ($year^{-1}$)	men		Distribution sampled using Markov Chain Monte Carlo methods.		14.4 (8.5, 22.9)	[17]
	women				14.4 (8.5, 22.9)	[17]
Proportion of population sexually active	men	16-19 years	506	208	0.709 (0.675, 0.741)	[18]
		20-24 years	467	45.8	0.911 (0.885, 0.934)	
		16-24 years	1090	234	0.823 (0.802, 0.843)	
	women	16-19 years	532	238	0.691 (0.658, 0.723)	[18]
		20-24 years	569	53.8	0.914 (0.890, 0.934)	

		16-24 years	1280	283	0.819 (0.799, 0.838)	
Proportion of incident infections which are asymptomatic	men		Calibrated to reproduce Natsal-3 prevalence		0.511 (0.264, 0.759)	[19]
	women				0.615 (0.468, 0.752)	

Supplementary Table 1: Distributions used as priors for model parameters. (For full methods, see Lewis et al.[20]) Test performance parameters are beta-distributed based on literature studies with parameters (α, β) as given, equal to one plus the numbers of positive and negative results in the study, respectively. The rates of spontaneous recovery and treatment seeking were sampled using MCMC methods (see main text). The proportion of the population who were sexually active is a beta distribution with parameters such that its central 95% credible interval matches the 95% confidence interval for the proportion estimated from Natsal-3. The proportion of incident infections which are asymptomatic was calibrated so that the sampled prevalence in 15-24-year-olds matched the Natsal estimates (see main text).

		Men		Women	
		15-19 years	20-24 years	15-19 years	20-24 years
2008-2009	Maximum	-0.350 (-0.772, -0.119)	-0.249 (-0.684, -0.043)	-0.640 (-1.056, -0.362)	-0.282 (-0.520, -0.141)
	Minimum	-0.355 (-0.786, -0.119)	-0.260 (-0.725, -0.049)	-0.641 (-1.056, -0.359)	-0.282 (-0.519, -0.140)
2009-2010	Maximum	-0.383 (-0.656, -0.189)	-0.284 (-0.547, -0.122)	-0.543 (-0.825, -0.339)	-0.206 (-0.335, -0.119)
	Minimum	-0.391 (-0.665, -0.197)	-0.288 (-0.552, -0.127)	-0.544 (-0.822, -0.340)	-0.206 (-0.337, -0.117)

Supplementary Table 2: Decrease in prevalence, in percentage points, inferred using maximum or minimum estimated tests and diagnoses, by sex, age group and annual period (2008-2009 and 2009-2010). All inferred values are provided as posterior median (95% credible interval).

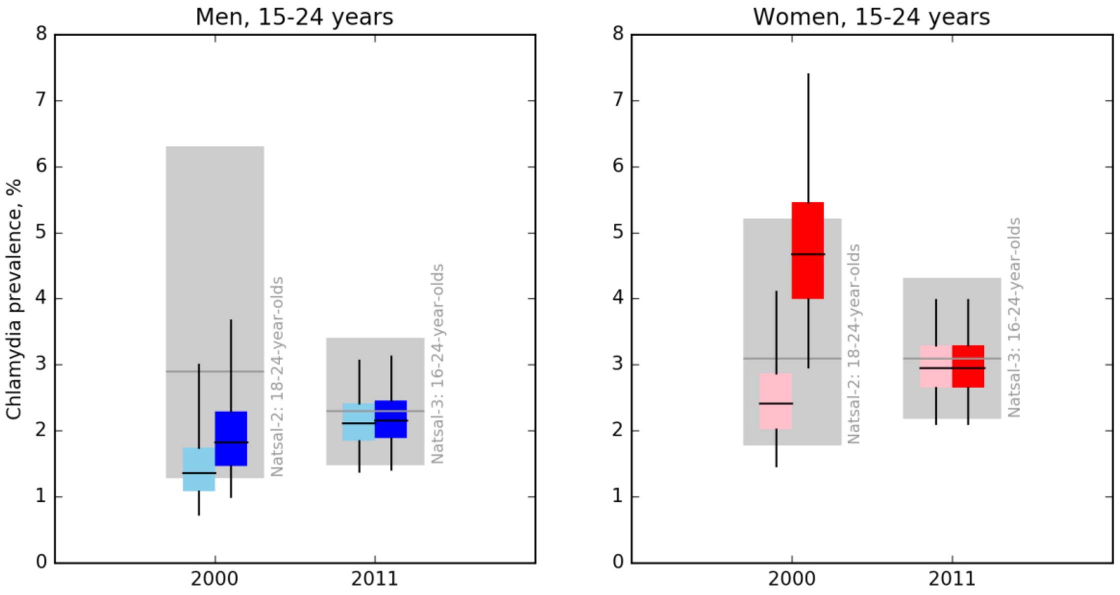

Supplementary Figure 1: Comparison between model-based (inferred) and survey-based (Natsal) prevalence estimates. Light- and dark-coloured boxes show estimates from the lower and upper bounds for the numbers of tests and diagnoses, respectively. The horizontal line indicates the median of each posterior distribution, the box the interquartile range, and the whiskers the 95% credible interval. The shaded bars show the 95% confidence intervals of the Natsal estimates, with the point estimate indicated by a horizontal line.

The plots show the good agreement between the population-based Natsal prevalence surveys and our model-based method, informed by surveillance data. For men in Natsal-2 the higher prevalence estimated from the survey vs the surveillance data is likely to be because only those aged 18+ years were included in Natsal-2, and prevalence in younger ages is much lower.

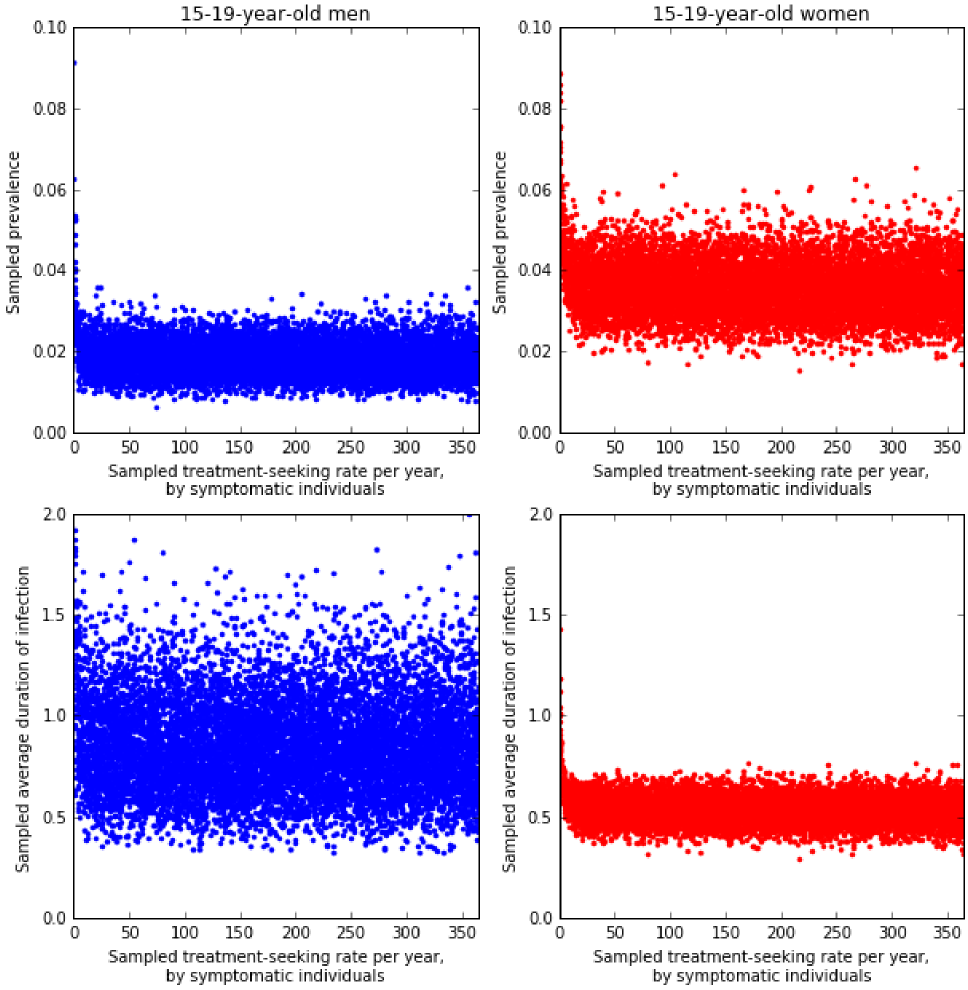

Supplementary Figure 2: Sensitivity of sampled prevalence and average duration of infection to sampled treatment-seeking rate by symptomatic individuals. The plots use samples for testing and diagnosis rates from distributions corresponding to 15-19-year-old men and women in 2012 (the year to which the model was calibrated). For each sample, shown as a dot, the x-coordinate indicates the treatment-seeking rate, sampled from a uniform distribution. The y-coordinate indicates the sampled prevalence (upper panels) or average duration of infection (lower panels).

Both inferred quantities are insensitive to the sampled treatment-seeking rate for all rates above ~5 per year, corresponding to an average time to treatment of <2 months. Hence, if the average time to treatment for symptomatic infections is less than two months, which seems likely in all ages and risk groups, the inferred results are insensitive to the rate of treatment seeking.

References

1. Horner P, Skidmore S, Herring A, et al. Enhanced Enzyme Immunoassay with Negative-Gray-Zone Testing Compared to a Single Nucleic Acid Amplification Technique for Community-Based Chlamydial Screening of Men. *J Clin Microbiol* 2005; **43**: 2065-2069.
2. Low N, McCarthy A, Macleod J, et al. Epidemiological, social, diagnostic and economic evaluation of population screening for genital chlamydial infection. *Health Technol Assess* 2007; **11**: 8.
3. Price MJ, Ades AE, De Angelis D, et al. Mixture-of-exponentials models to explain heterogeneity in studies of the duration of *Chlamydia trachomatis* infection. *Stat Med* 2013; **32**: 1547-1560.
4. Handsfield HH, Alexander R, Wang SP, Pedersen AHB, Holmes KK. Differences in the therapeutic response of chlamydia-positive and chlamydia-negative forms of nongonococcal urethritis. *J Amer Ven Dis Assoc* 1976; **2**: 5-9.
5. Prentice MJ, Taylor-Robinson D, Csonka GW. Non-specific urethritis: A placebo-controlled trial of minocycline in conjunction with laboratory investigations. *Brh J Vener Dis* 1976; **52**: 269-275.
6. Johannisson G, Sernryd A, Lycke E. Susceptibility of *Chlamydia trachomatis* to antibiotics in vitro and in vivo. *Sex Transm Dis* 1979; **6**: 50-57.
7. Paavonen J, Kousa M, Saikku P, Vartiainen E, Kanerva L, Lassus A. Treatment of nongonococcal urethritis with trimethoprim-sulphadiazine and with placebo: A double-blind partner-controlled study. *Br J Vener Dis* 1980; **56**: 101-4.
8. Joyner JL, Douglas JM, Foster M, Judson FN. Persistence of *Chlamydia trachomatis* infection detected by polymerase chain reaction in untreated patients. *Sex Transm Dis* 2002; **29**: 196-200.
9. Geisler WM, Wang C, Morrison SG, Black CM, Bandea CI, Hook EW III. The natural history of untreated *Chlamydia trachomatis* infection in the interval between screening and returning for treatment. *Sex Transm Dis* 2008; **35**: 119-123.
10. Stamm WE, Cole B. Asymptomatic *Chlamydia trachomatis* urethritis in men. *Sex Transm Dis* 1986; **13**: 163-165.
11. van den Brule AJC, Munk C, Winther JF, et al. Prevalence and persistence of asymptomatic *Chlamydia trachomatis* infections in urine specimens from Danish male military recruits. *Int J STD AIDS* 2002; **13**: 19-22.
12. Rahm VA, Belsheim J, Gleerup A, Gnarpe H, Rosen G. Asymptomatic carriage of *Chlamydia trachomatis* - a study of 109 teenage girls. *Eur J Sex Transm Dis* 1986; **3**: 91-94.
13. Sørensen JL, Thranov I, Hoff G, Dirach J. Early- and late-onset pelvic inflammatory disease among women with cervical *Chlamydia trachomatis* infection at the time of induced abortion--a follow-up study. *Infection* 1994; **22**: 242-246.
14. McCormack WM, Alpert S, McComb DE, Nichols RL, Semine DZ, Zinner SH. Fifteen-Month Follow-up Study of Women Infected with *Chlamydia trachomatis*. *N Eng J Med* 1979; **300**: 123-125.
15. Morré SA, van den Brule AJC, Rozendaal L, et al. The natural course of asymptomatic *Chlamydia trachomatis* infections: 45% clearance and no development of clinical PID after one-year follow-up. *Int J STD AIDS* 2002; **13**: 12-18.
16. Molano M, Meijer CJLM, Weiderpass E, et al. The natural course of *Chlamydia trachomatis* infection in asymptomatic Colombian women: A 5-year follow-up study. *J Infect Dis* 2005; **191**: 907-916.

17. Mercer CH, Sutcliffe L, Johnson AM, et al. How much do delayed healthcare seeking, delayed care provision, and diversion from primary care contribute to the transmission of STIs? *Sex Transm Infect* 2007; **83**: 400-405.
18. Johnson A, London School of Hygiene and Tropical Medicine. Centre for Sexual and Reproductive Health Research and NatCen Social Research. *National Survey of Sexual Attitudes and Lifestyles, 2010-2012*. Colchester, Essex: UK Data Archive, September 2015. SN: 7799, http://dx.doi.org/10.5255/UKDA-SN-7799-1.
19. Sonnenberg P, Clifton S, Beddows S, et al. Prevalence, risk factors, and uptake of interventions for sexually transmitted infections in Britain: findings from the National Surveys of Sexual Attitudes and Lifestyles (Natsal). *Lancet* 2013; **382**: 1795-1806.
20. Lewis J, White PJ. Estimating local chlamydia incidence and prevalence using surveillance data. *Epidemiol* 2017; **28**: 492-502.
